# PRC1 Protein Subcomplexes Architecture: Focus on the Interplay between Distinct PCGF Subunits in Protein Interaction Networks

**DOI:** 10.3390/ijms25189809

**Published:** 2024-09-11

**Authors:** Nayla Munawar, Kieran Wynne, Giorgio Oliviero

**Affiliations:** 1 Department of Chemistry, College of Science, United Arab Emirates University, Al Ain 15551, United Arab Emirates; 2 Conway Institute of Biomolecular and Biomedical Research, University College Dublin, D04 C1P1 Dublin, Ireland; kieran.wynne1@ucd.ie; 3 Systems Biology Ireland, School of Medicine, University College Dublin, D04 C1P1 Dublin, Ireland

**Keywords:** polycomb-group proteins (PcG), PRC1, PCGF, epigenetics, immunoprecipitation, mass spectrometry, chromatin regulation, histone modifications, NT2 cells

## Abstract

The six PCGF proteins (PCGF1-6) define the biochemical identity of Polycomb repressor complex 1 (PRC1) subcomplexes. While structural and functional studies of PRC1 subcomplexes have revealed their specialized roles in distinct aspects of epigenetic regulation, our understanding of the variation in the protein interaction networks of distinct PCGF subunits in different PRC1 complexes is incomplete. We carried out an affinity purification mass spectrometry (AP-MS) screening of three PCGF subunits, PCGF1 (NSPC1), PCGF2 (MEL18), and PCGF4 (BMI1), to define their interactome and potential cellular function in pluripotent human embryonal carcinoma cell “NT2”. The bioinformatic analysis revealed that these interacting proteins cover a range of functional pathways, often involved in cell biology and chromatin regulation. We also found evidence of mutual regulation (at mRNA and protein level) between three distinct PCGF subunits. Furthermore, we confirmed that the disruption of these subunits results in reduced cell proliferation ability. We reveal an interplay between the compositional diversity of the distinct PCGF containing PRC1 complex and the potential role of PCGF proteins within the wider cellular network.

## 1. Introduction

Chromatin accessibility reflects the degree to which nuclear macromolecules physically compact DNA into a small volume within the nucleus. It is determined by the occupancy and topological organization of nucleosomes as well as other chromatin-binding factors that occlude access to DNA [1]. Chromatin-binding factors cooperatively regulate gene expression through the alteration of the chromatin architecture. A class of multi-heterooligomeric protein complexes known as chromatin remodelers can rearrange the accessible or permissive chromatin conformation through their enzymatic subunits. Several chromatin-remodeling protein complexes including Polycomb repressive complexes (PRC) regulate chromatin conformation and gene expression by post-translational modification (PTM) of the N-terminal tail regions of the histone proteins [2,3,4]. PRC complexes maintain heterochromatin and regulate gene expression during several biological processes like mammalian development, pluripotency, and carcinogenesis [5,6,7,8]. Abnormal PRC protein expression and/or mutation can lead to impaired signaling, which inhibits tumor suppressor activity or promotes proto-oncogene activity, leading to a loss of cell identity [9].

PRC complexes assemble in two major configurations: Polycomb repressive complex 1 (PRC1) is an E3 ubiquitin ligase that mono-ubiquitylates histone H2A at lysine 119 (H2AK119ub1), while Polycomb repressive complex 2 (PRC2) houses a methyltransferase that can mono-, di-, and tri-methylate histone H3 at lysine 27 (H3K27me1, H3K27me2, and H3K27me3) [8,9]. Some non-histone substrates (e.g., STAT3, RORα) can also be methylated [10]. The core of the PRC2 complex is composed of four proteins: EZH1/2, EED, SUZ12, and RBAP46/48 [11,12]. However, accessory PRC subunits have also been described such as AEBP2, JARID2, PCL1 (PHF1), PCL2 (MTF2), and PCL3 (PHF19) [12]. Some combinations of these accessory proteins are exclusively mutual, for example, JARID2 exists with most of the other accessory proteins [13,14].

The biochemical analysis of PRC1 has revealed diverse forms of these multiprotein complexes. In addition to the core E3 ligase and a RING1 protein (RING1A or RING1B), different PRC1 complexes contain one of six distinct Polycomb group RING finger proteins (PCGF1–6) exclusively [8,15]. These, PCGF1, PCGF2, PCGF3, PCGF4, PCGF5, and PCGF6 are also known as NSPC1, MEL18, RNF3, BMI1, RNF159, and MBLR, respectively. In addition, numerous other proteins are also associated with PRC1 complexes impacting their function in a distinct way. PRC1 complexes can be classified into canonical or non-canonical forms (cPRC1 and ncPRC1) [8]. Both complexes mediate histone H2A monoubiquitination via the E3 ubiquitin ligase component RING1A/B. Characteristically, cPRC1 complexes contain RING1A-PCGF2 and RING1A-BMI1 with thew E3 ligase [8,9]. In addition, cPRC1 complexes contain CBX (chromobox) proteins that target the complex trimethylated lysine 27 on histone 3 (H3K27me3) and are associated with chromatin condensation events [9,16]. On the other hand, ncPRC1 complexes contain other four PCGFs, PCGF1, PCGF3, PCGF5, and PCGF6, in addition to subunits having dedicated DNA binding domains. In ncPRC1 complexes, RYBP or YAF2 recognize H2AK119ub1, and are therefore linked to stronger ubiquitination activity [8].

All subsets of PRC1 complexes contain a heterologous collection of subunits with diverse functions [9,11,12]. Lysine-specific demethylase 2B (KDM2B), a PRC1-PCGF1 (PRC1.1) component, is responsible for the recruitment of PRC1.1 to a subset of CGI (CpG islands) promoters [17,18]. In mouse embryonic stem cells, PRC1-dependent H2AK119ub1 leads to the recruitment of PRC2 and H3K27me3 to effectively initiate a Polycomb domain. This activity is relatively restricted to the ncPRC1 variant PCGF1–PRC1 complex that recognizes non-methylated DNA in CGIs by the CxxC-ZF domain of KDM2B [17]. This contributes to histone H2A lysine 119 ubiquitylation and gene repression [17,19].

Hence, much research related to Polycomb-mediated gene repression has employed mouse embryonic stem cells (mESCs), a powerful yet accessible model [20,21,22,23,24,25]. Pluripotent embryonic carcinoma cell lines, such as NTera-2/cloneD1 (NT2), are an important tool for studying pluripotent and stem cell-like differentiation programs in a human model [26,27,28]. Upon treatment with retinoic acid (RA), NT2 cells can be induced to differentiate into neuron-like cells, which display a variety of neurotransmitter phenotypes [29,30,31].

In our previous investigation on ncPRC1, the PCGF1–PRC1 complex showed its function in maintaining the embryonic cell fate by interacting with pluripotency markers such as OCT4, NANOG, and DPPA4 [32]. Here, we extended our investigation to other cPRC1–PRC1 complexes that contain PCGF subunits such as PCGF2 and PCGF4/BMI1 on the basis of their sequence homology to define their specific interactome in native NT2 cells. Despite all three PCGFs sharing a certain degree of protein homology, the overall common interactome is only 10%. The cell viability assay showed a unique function of PCGF4/BMI1 in cell proliferation compared to other PCGF proteins. Our findings highlight that approximately 30% of protein sequence differences between PCGF2 and PCGF4/BMI1 permit the recruitment of a unique interactome for these subunits that consequently affects the biological role of their corresponding two cPRC1 complexes.

## 2. Results

### 2.1. A Physical Interaction Screen for PRC1 Components Purified under Endogenous Conditions

**Figure 1:**
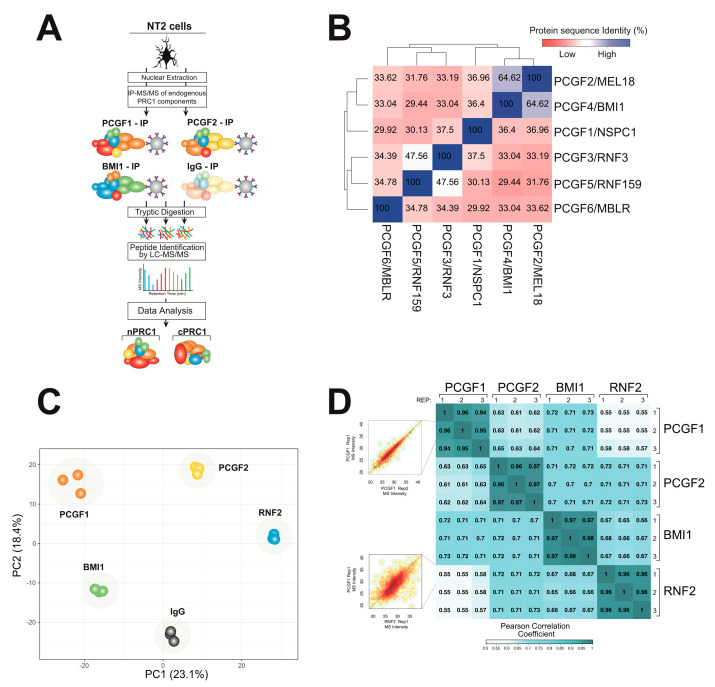
A physical interaction screen for the PRC1 components. (**A**) Four subunits of PRC1 subcomplexes were purified under endogenous conditions using immunoprecipitation (IP). Physically interacting proteins were identified and quantified using Orbitrap mass spectrometry. (**B**) Amino acid homology between all six human PCGF proteins was compared using Clustal Omega. (**C**) Sensitivity and reproducibility of the biological replicate and individual IP experiments were compared by plotting a matrix of pairwise Pearson correlation coefficients. (**D**) The physical interactomes of each IP experiment (including biological replicates) were compared using principal component analysis.

An affinity immunoprecipitation approach, combined with high-resolution mass spectrometry (AP-MS), has been used to identify physical protein interactions of PRC1 components in NT2 cells under the endogenous condition to avoid artifacts that could arise from the overexpression of a protein (Figure 1A) [33,34]. Nuclear lysates were immunoprecipitated with α-PCGF1, α-PCGF2, and α-PCGF4/BMI1 using an α-Rabbit IgG antibody as a negative control. Since RNF2 is known to be an interactor of all PCGF members, we used anti-RNF2/RING1B as a positive control. Soluble peptides from each lysate were obtained by trypsin digestion in situ on agarose beads. The peptides were analyzed by mass spectrometry, and the raw data were used to identify and quantify the relative abundance of the proteins using the MaxQuant platform [35].

A comparison of the amino acid sequences of the PCGF variants using multiple alignments [36,37] showed the highest sequence homology between PCGF2 and PCGF4/BMI1 (Figure 1B). Two protein domains are shared across all PCGF subunits: the RING finger and RAWUL domains [37,38]. These domains likely play an important role during the assembly of the PRC1 complexes [39,40]. Interestingly, PCGF2 and PCGF4/BMI1 also contain extended C-terminal regions that may be associated with protein disorder [37,38]. A weaker level of homology, around 30%, was observed among other PCGF variants (Figure 1B). The high sequence similarity between PCGF2 and PCGF4/BMI1 led us to hypothesize that homolog proteins may share a large number of interactor proteins and subsequently affect the assembly across the canonical and not canonical PRC1 complex.

Therefore, next, we investigated the relationship between the amino acid sequence and interactome similarity of different PCGF proteins. The sensitivity and reproducibility of the experimental approach were confirmed using biological replicates. Approximately 0.95, the average Pearson correlation between each biological AP-MS replicate indicated a high degree of experimental reproducibility (Figure 1C). Between the rest of the AP-MS, a reasonable correlation (~0.6–0.7) was observed for the three PGCF proteins, while the single non-PCGF protein analysis (RNF2) showed a slightly lower correlation (~0.5), as expected.

In order to evaluate the overall dataset, we used principal component analysis (PCA) using the summed peptide mass spectrometry signal intensities for each identified protein as input (‘label free quantitation’ value from MaxQuant [34,41] (Figure 1D). Reassuringly, each immunoprecipitation experiment was well separated, while the biological experimental replicates were located close together, showing the robustness of our strategy.

### 2.2. Affinity Purification (AP)-MS Screening Reveals Common and Distinct Interactomes among PRC1–PCGF Complexes

**Figure 2:**
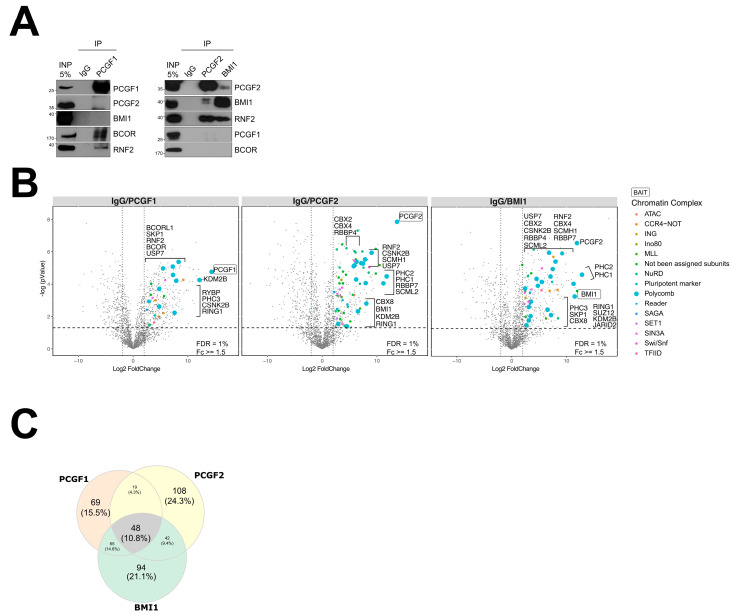
AP-MS screening revealed common and distinct interactomes among PRC1 component proteins. (**A**) The specificity of the antibodies used in the PCGF1, PCGF2, and PCGF4/BMI1 immunoprecipitations was confirmed using Western blotting. (**B**) The set of identified proteins for each immunoprecipitation experiment was projected onto volcano plots to identify statistically robust hits. (**C**) Venn diagram showing the range of overlap among the physical interactomes of PCGF1, PCGF2, and PCGF4/BMI1 as well as subsets uniquely identified for each bait.

To assess the distinctive PCGF subunit interactomes, we compared the protein abundance in samples immunoprecipitated using α-PCGF1, α-PCGF2, α- PCGF4/BMI1, and α-RNF2 (RING1B) to samples immunoprecipitated using IgG as a negative control (Supplementary Materials). The specificity and effectiveness of the antibodies were confirmed using co-immunoprecipitation experiments (Figure 2A and Supplementary Material Figure S1B). Each antibody immunocaptured its cognate bait protein, while all three PCGF baits co-precipitated the expected common PRC1 complex subunit RING1A and RING1B/RNF2, an E3 ubiquitin ligase for H2AK119ub and essential component of PRC1 in mammals [41,42] (Figure 2A).

To support our strategy, we investigated the RNF2 interactome as PRC1 quality control. In line with previous PRC1 interactome analyses [43,44], RNF2 was immunoprecipitated with all six PCGF subunits (Supplementary Material Figure S1B). Volcano plots were used to project each protein onto a chart showing enrichment in each immunoprecipitation experiment relative to the IgG control (*x*-axis) versus the significance of that finding based on the *t*-test (*y*-axis) (Figure 2B). A total of 48 interactions were shared across all PCGF proteins (Figure 2C). PCGF2 and PCGF4/BMI1 shared 42 interactions, while PCGF1 and PCGF2 shared 19 potential co-precipitated candidates, and PCGF1 and PCGF4/BMI1 had 65 co-precipitated proteins in common. In general, the protein interactions observed in the NT2 cells in our study correlated well with some already reported observations [43,45], suggesting that the composition of PRC1 complexes is reasonably conserved despite different cellular contexts (Supplementary Material Figure S1C). In the current study, we aimed to elucidate the PCGF-PRC1 architecture in the presence of auxiliary subunits, classified as either non-canonical or canonical PRC1 by employing affinity protein purification. In line with a certain degree of protein homology divergence across all PCGF subunits, we report that the BCOR component of non-canonical PRC1 complexes was only precipitated with anti-PCGF1 but not with anti-PCGF2 and anti-PCGF4/BMI1, showing a very specific interaction of PCGF1 with the BCOR protein. Likewise, PCGF2 and PCGF4/BMI1 interactomes contained the chromo-domain protein CBX2, CBX4, and CBX8, which are signatures of canonical PRC1 complexes. Overall, we observed that the PCGF interactomes contained a heterogeneous collection of subunits, sometimes in a sub-stoichiometric manner, demonstrating the differences inherent in the PRC1 architecture.

Since the native PCGF interactome had not been completely elucidated, we compared our data with previously published data by Gao et al. [43] and Hauri et al. [44] by using a Venn diagram to determine common and unique PRC1 features obtained by diverse protein characterization strategies (Supplementary Material Figure S1C). We found that all PCGF-candidates co-precipitated, as reported by Gao et al. [43] and Hauri et al. [44], were also identified in the current study. Very importantly, we also observed almost 200 interacting candidates across PCGF1, PCGF2, and PCGF4/BMI1, interactomes that have not been described yet (Supplementary Materials). The identification of new co-precipitated candidates in our study might be the result of a change in experimental and analytical strategies compared to the studies performed by other researchers [44,45]. It is also important to mention that PRC1 complexes are highly dynamic structures that evolve in tandem with the progression between cell states [46].

### 2.3. Relative Abundance Estimation and Molecular Mass of the Isolated PRC1 Complexes

Many of the interactions we identified in this study are components of other chromatin remodeler complexes (Figure 2B and Supplementary Material Table S1). By retrieving the most recent depository of the chromatin remodeling complex [47,48,49], we specifically detected the following: Ada2a-containing (ATAC) ATAC; carbon catabolite repression (CCR4) negative; inhibitor of growth (ING); mixed-lineage leukemia (MLL); nucleosome remodeling and deacetylases (NuRD); Spt-Ada-Gcn5 acetyltransferase (SAGA); SET domain-containing protein (SET); histone deacetylase complex subunit (SIN3A); SWItch/Sucrose Non-Fermentable (SWI/SNF); and the general transcription factor IID (TFIID). Our results are in line with Hauri et al. [44], who reported PRC1 and PRC2 co-purified with several chromatin remodeling subunits encompassing MLL, NSL, the ADA2/GCN5/ADA3 transcription activator, NURF, NURD, and SIN3 complexes [44].

In addition, we distinguished unique and different chromatin remodelers that are not yet assigned as PRC1 co-precipitated proteins such as a CCR4–NOT complex uniquely co-purified with PCGF1. Very interestingly, we observed that the CNOT1 and CNOT4 subunits that interacted with all PCGF bait proteins also included RNF2 bait. CNOT1 and CNOT4 are involved in E3 ligase activity and promote histone ubiquitination [50,51], which explained a possible reason for interaction with the PCGF subunits and RNF2. Moreover, we identified other subunits related to the BAF complex such as ARID2, ATRX, BRD7, SMARCB1, SMARCA4, SMARCC1, SMARCC2, SMARCD1, SMARCD2, and SMARCE1, which were purified with our bait proteins [52,53].

**Figure 3:**
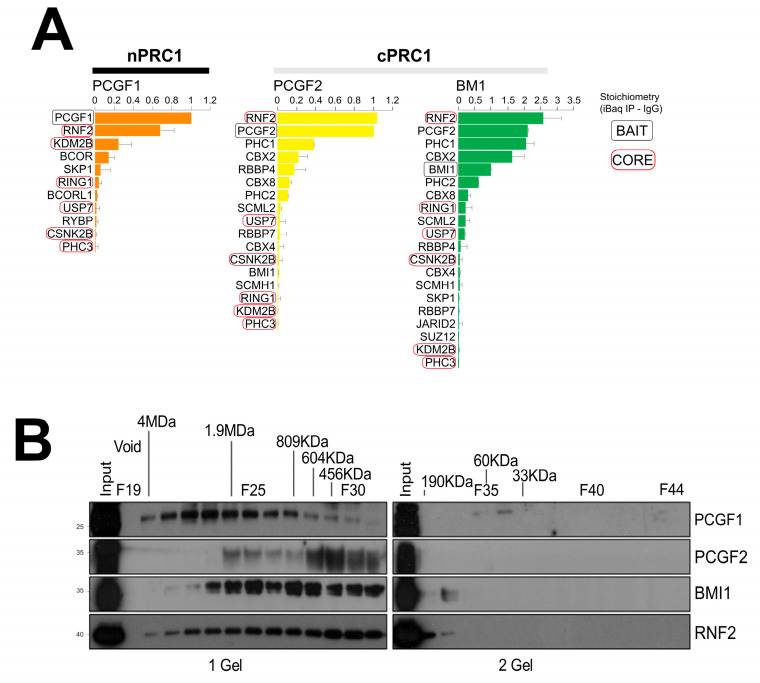
Stoichiometry and molecular mass of the isolated PRC1 complexes. (**A**) The relative abundance estimation contributions of individual subunits to each IP was estimated using the peptide signal intensity adjusted for protein size. Relative quantities were normalized to the signal recorded for bait peptides. (**B**) Nuclear lysate fractions separated by gel filtration chromatography were analyzed on SDS-PAGE and probed using antibodies against PCGF1, PCGF2, PCGF4/BMI1, and RNF2.

The mechanism by which BAF complex disengagement leads to the Polycomb repressor complex-driven re-establishment of heterochromatin signatures associated with gene repression is still not defined [53]. However, we did not perform any further validation studies to confirm these novel BAF complex candidates and PRC1 interactions due to the lack of highly specific antibodies against BAF components. Since PRC1 is itself a high molecular weight (MW) multiprotein assembly, and the potential interactors identified here are components of diverse multiprotein assemblies, we just focused on analyzing the physical form of only the PRC1 complexes isolated during these experiments. First, we estimated the stoichiometry of subunits present in each PRC1 complex by dividing the normalized mass spectrometry intensity signal for each protein (LFQ) by the protein MW (iBAQ score) [54] (Figure 3A). Core members of the PRC1 complex (such as RNF2, RING1A, KDM2B, USP7, CSNK2B, and PHC3) that are shared among all three PCGF–PRC1 interactome complexes were present in approximately equal stoichiometry. PCGF2-PRC1 and PCGF4/BMI1-PRC1 variant interactomes exhibited a common stoichiometric pattern profile and shared canonical PRC1 subunits including the following chromobox proteins: PHC1, PHC2, CBX2, CBX4, CBX8, SCML2, SCMH1, RBBP4, and RBBP7. To investigate the molecular mass of the isolated complexes, nuclear protein lysate from NT2 cells was separated by size exclusion chromatography and the fractions were probed using antibodies against PCGF1, PCGF2, PCGF4/BMI1, and RNF2 (Figure 3B). All four PRC1 component proteins were found to be present in high-mass complexes. These complexes varied greatly in size, from 200 KDa to 4 MDa. The size exclusion experiments confirmed that the high mass complexes that contained PCGF2 and PCGF4/BMI1 were close in size and largely overlapped, while the PCGF1 profile seemed to belong to a higher mass above the 2 MDa range complexes (Figure 3B).

### 2.4. Functional Enrichment of PCGF Interactome Map to Multiple Pathways

**Figure 4:**
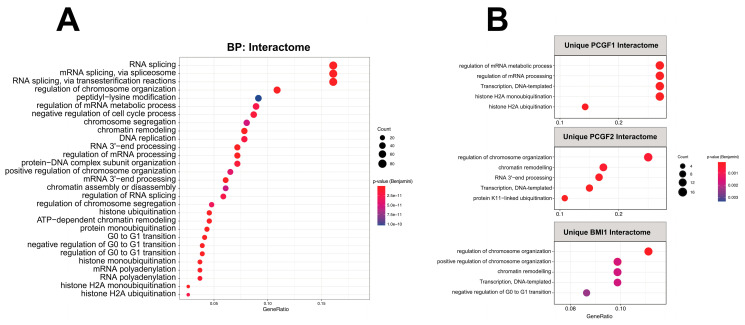
Functional enrichment of PCGF interactomes mapped to multiple pathways. Gene Ontology (GO) analysis was used to identify enriched pathway components in the overall (**A**) and individual PCGF interactomes (**B**). Categories were sorted by *p*-value (Spearman’s rank correlation coefficient), while the dot size represents the number of proteins corresponding to the source pathway.

Next, we used the Gene Ontology (GO) analysis (“BP”, “biological process”) to investigate whether the observed interactomes were associated with particular molecular pathways. We created a comprehensive overview mapping of the pathways associated with the PCGF interactome by analyzing the whole PCGF interactome obtained during this study. The functional categories are displayed in a dot plot cluster and represent the significant biological process enriched (Figure 4). Overall, five main categories were found to be significantly enriched: “mRNA splicing”, “regulation of chromosome organization”, “histone ubiquitination”, “regulation of G0 to G1 transition”, and “histone H2A monoubiquitination”. We performed a similar analysis among the distinctive PCGF interactomes to assess the unique pathways that may affect PRC1 organization through their unique PCGF features (Figure 4B). The PCGF2 and PCFG4/BMI1 interactomes exhibited similar biological functional properties including common biological annotations such as “regulation of chromosome organization”, “transcription, DNA-templated”, and “chromatin remodeling”. We also observed cell cycle-related terms such as “negative regulation of G0 to G1 transition” as uniquely enriched pathways in the PCGF4/BMI1 interactome, while “histone ubiquitination” categories were shared between the PCGF1 and PCGF2 interactomes. Overall, our GO analysis is in line with previous studies and confirmed known Polycomb-related pathways such as “regulation of chromosome organization” and “chromatin remodeling”. In addition, we observed distinctive biological pathways relative to each PCGF interactome.

### 2.5. The Role of PCGF Subunits in NT2 Cells and Mutual Regulation of PCGF Variants

**Figure 5:**
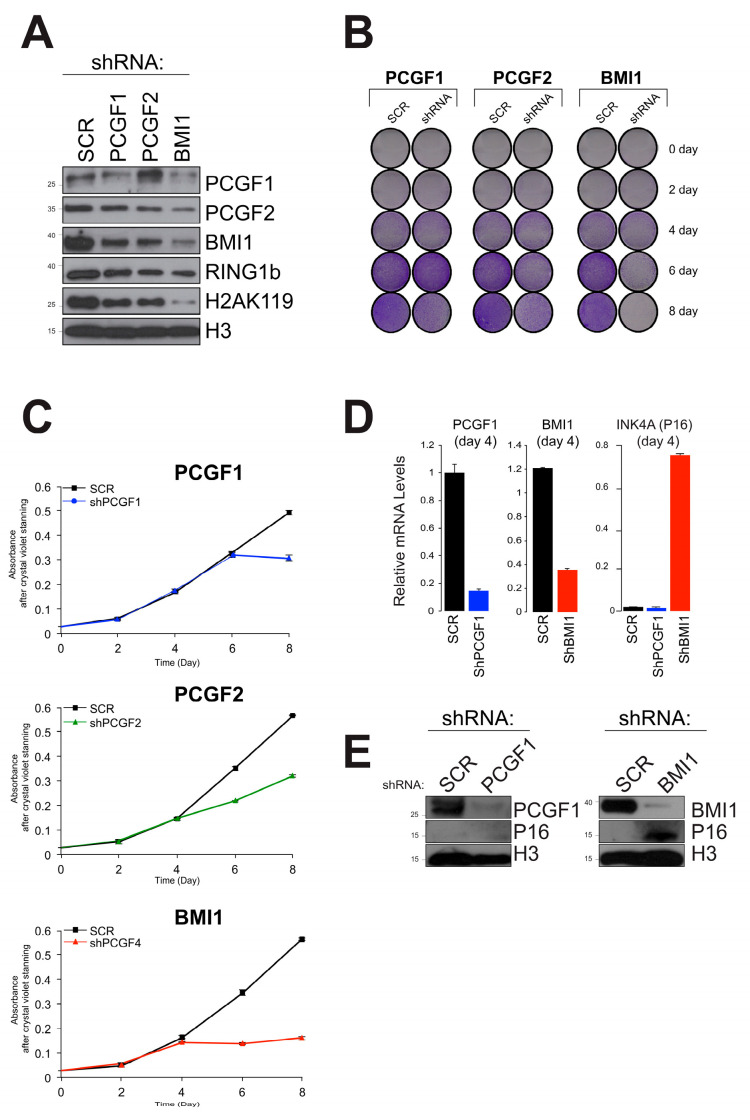
Functional enrichment of PCGF interactomes mapped to multiple pathways. (**A**) Western blotting was used to confirm the successful partial knockdown of protein expression for PCGF1, PCGF2, and PCGF4/BMI1. Ubiquitination of histone H2A K119 was also reduced. (**B**) Cell proliferation was quantified using the crystal violet assay. (**C**) This assay allowed a comparison of the kinetics for each knockdown, with PCGF4/BMI1 showing a more pronounced reduction in the proliferation rate than PCGF1 or PCGF2. (**D**) The INK4A-P16 marker of cellular senescence was upregulated by the depletion of PCGF4/BMI1 but not PCGF1. (**E**) Western blot confirmed the regulation of INK4A-P16 by the deletion of PCGF1 and PCGF4/BMI1.

In order to assess the functional effect of disrupting PCGF expression in NT2 cells, we carried out knockdown screens for the PCGF subunits. Successful knockdown of each PCGF variant was achieved using the shRNA method (Figure 5A, Supplementary Material Figure S2). As expected, global levels of H2BK119ub were reduced, most prominently for PCGF4/BMI1, in line with previous reports [55]. Furthermore, the levels of PCGF4/BMI1 mRNA itself were reduced following the knockdown of PCGF2 and vice versa. This suggests that PCGF2 and PCGF4/BMI1 may influence their regulation at both the transcriptional and protein levels. This corroborates previous evidence of a synergistic requirement for these PcG proteins in the maintenance of Hox gene expression during early mouse development [56]. Mouse embryos deficient in PCGF2 and PCGF4/BMI1 exhibit similar posterior transformations of the axial skeleton and display severe immune deficiency [56]. Conversely, some minor effects of PCGF1 protein expression alteration seem to be detected during PCGF2 downregulation. These results suggest an auto-regulatory activity among many, or even all, PCGF genes.

The crystal violet assay was performed to observe cell viability by depleting PCGF subunits. The highest reduction in cell growth rate was observed after four days of disruption of PCGF4/BMI1 expression. A similar trend, although slightly less pronounced, was observed for PCGF2 depletion, while in the PCGF1 samples, the cell proliferation rate did not change (Figure 5B,C).

We subsequently generated a PCGF lentivirus for the quantitative assessment of gene knockdown expression in NT2 cells (Figure 5C). The observation that PCGF4/BMI1 can influence the cell growth rate raises the possibility of a mechanism related to senescence. In line with a previous study, regulatory mechanisms yielded by PCGF4/BMI1 expression control the cell cycle through the regulation of the Ink4a/Arf locus [57,58]. To confirm this, we measured the mRNA and protein levels of p16, a senescence marker [59,60], at the protein and transcriptome levels, respectively. Interestingly, only PCGF4/BMI1 was found to influence cell viability through p16 expression (Figure 5D,E). This may suggest that other PCGF proteins can influence cell growth via independent, non-senescent pathways.

## 3. Discussion

In the last decade, the PRC1 complex has been intensively investigated [22,23,61,62] to screen its precise role in chromatin remodeling and other cell events including cancer. To date, a detailed dissection of the PRC1 assembly composition indicates the presence of six different PRC1 variants. Each PRC1 variant exhibits a different PCGF subunit, indicating that PRC1 complexes contain mutually exclusive homologs of the PCGF protein, resulting in six distinct PRC1 subcomplexes (PRC1.1 to PRC1.6) [15,43,63]. Despite all six PRC1 subcomplexes having different subunits reflecting diverse functional properties, it is still unclear how PCGF proteins can influence the PRC1 architecture.

Recently, the role of the PRC1-6 subcomplexes has been studied by combining the in-house development of highly specific PCGF1-6 antibodies with the generation of KO mESC lines depleted for all six PCGF proteins [15,63]. The genome-wide occupancy of all PRC1 subcomplexes was mapped to determine their functional control in pluripotent cell modeling. The results suggest that the activities of PCGF1 and PCGF2 are strongly linked to transcriptional repression and display extensive functional overlap [15].

For over two decades, the role of Polycomb-mediated gene repression has been mostly dissected in mouse embryonic stem cells (mESCs), which is considered a “gold standard” model for epigenetics research [20,24,25]. The focus on this method has limited Polycomb characterization in other cell models such as cell types that reflect the tumorigenesis environment or exhibit cancer genotype and heterogeneity. Moreover, recent advances have shown that Polycomb proteins form distinct multi-protein complexes in different cellular environments such as in early development, adult tissue maintenance, and cancer. This dynamic diversity mainly influences target gene repression and the inheritance of memory. More recently, Polycomb dissection has taken a significant step forward in understanding their role as epigenetic markers that could potentially cause age-dependent changes [64]. In our study, we evaluated the role of PCGF-PRC1 organization in NT2 cells, a cell therapy model for the investigation of human neurogenesis, regeneration, and drug screening that has not been elucidated yet [26,27,28].

Previous studies identified two domains shared across all PCGF proteins: RING finger and RAWUL [37,38,39], which play an important role in defining PRC1 composition.

Following our previous investigation on ncPRC1 and the PCGF1–PRC1 complex in maintaining embryonic cell fate [32], we investigated the physical interactomes of only the PCGF1/NSPC1, PCGF2/MEL18, and PCGF4/BMI1 subunits in PRC1 complexes during this study. Due to the lack of commercially available antibodies against PCGF3, PCGF5, and PCGF6, we did not attempt any further investigations on these PCGFs. Moreover, we observed low protein expression levels of PCGF3, PCGF5, and PCGF6 by protein dynamic range analysis in our NT2 cell model (Supplementary Material Figure S1A), which hinders vibrant investigation under native conditions, providing us with another reason for not including these PCGF variants in this study.

Therefore, PCGF1, PCGF2, and PCGF4/BMI1, which show a high degree of protein sequence homology, were expressed in NT2 cells under native conditions. We favored an endogenous immunoprecipitation approach for analyzing the PCGF1, PCGF2, and PCGF4/BMI1 subunits to characterize the behavior of the PCGF-PRC1 variant in a manner as close to the native cell condition as possible, in particularly avoiding artifacts arising from the exogenous expression affinity-tagged form. Previously, the TAP-tag strategy was used to target PCGF proteins and their surrounding interactome [43,45]. Although the TAP-tag has been shown to be an efficient strategy, several limitations arise that interfere with protein function, location, and complex formation. This is of particular concern when proteins share a high level of sequence homologies such as in the case of PCGF2 and PCGF4/BMI1.

Therefore, we applied affinity purification-mass spectrometry (AP-MS) to dissect the PCGF-PRC1 variant assembly and distinguish the individual interactors that associated preferentially in only one PCGF-PRC1 variant, in two PCGF-PRC1 variants, or in all three PCGF-PRC1 variants. We observed a significant degree of common interactions among each purified PCGF, consistent with previous reports [43,44]. This suggests that the composition of PRC1 complexes is reasonably conserved, despite a difference in cellular contexts. Additionally, we reported that our PCGF interactomes exhibited a similar protein network previously observed in Drosophila embryos [65]. As expected, the PcG complexes exhibited an evolutionarily conserved core group of subunits with conserved function to maintain homeotic genes in a silenced state during development [66].

However, our study also found novel co-precipitated candidate proteins. The identification of over 200 potential interacting candidates in the PCGF1, PCGF2, and PCGF4/BMI1 interactome in this study (Figure 1 and Figure 2) could be attributed to our different experimental and analytical strategies compared to those in previous studies. For example, the intrinsic quality of the TAP-tag strategy that has been employed in other studies may affect the affinity binding efficiency. Therefore, a relatively low efficiency of purification can be observed. Furthermore, ectopic protein expression following tandem affinity purification was unable to isolate and identify interacting proteins in 22% of the purified tagged proteins in the yeast proteome [34]. On the other hand, the AP-MS approach under native conditions may explain the large number of novel chromatin remodeling subunits detected in our study in comparison with previous reports.

We then dissected the distinctive functional biological of the PCGF-PRC1 interactome through Gene Ontology (GO) analysis to investigate common and unique pathways relative to the chromatin environment. We reported cell cycle-related terms such as “negative regulation of G0 to G1 transition” as uniquely enriched pathways in the PCGF4/BMI1 interactome, while, as expected, “histone H2A ubiquitination” related categories were shared across all three PCGFs.

Finally, we explored the role of PCGF subunits in the gene regulation of NT2 cells using knockdown screening against the cognate PCGF subunits. We observed through the cell viability assay that PCGF4/BMI1 uniquely affects cell proliferation, rather than the other PCGF auxiliaries. Despite PCGF2 and PCGF4/BMI1 sharing amino acid sequences and relative overlapping interactomes, only PCGF4/BMI1 directly regulated the expression of the Ink4a/Arf gene locus. This result is in line with the observations by Morey et al. [46], where the gene expression between PCGF2 and PCGF4/BMI1 was alternatively correlated during mESCs differentiation. Moreover, pluripotent mESCs did not express detectable levels of PCGF4/BMI1 in either the protein or transcriptional levels, and forced PCGF4/BMI1 expression had no obvious influence on mESC self-renewal [67]. Additionally, the combinatorial depletion of PCGF2 and PCGF4/BMI1 expression exhibited severe growth retardation, accelerated apoptosis, and defects in the maintenance of stable gene expression during early development in mouse cells [56]. To date, the roles of PCGF2 and PCGF4/BMI1in cell proliferation, differentiation, and embryogenesis have been deeply investigated. For example, PCGF2 is a target of the protein kinase AKT [68]. AKT phosphorylates PCGF2 to disrupt the interaction between PCGF2 and other PRC1 members that cause tumorigenesis in breast cancer [58,69]. Moreover, PCGF2 has been found to be essential for ESC differentiation into early cardiac-mesoderm precursors and exhibits a distinctive PCGF2-PRC1 activity to control the expression of the negative regulators of the BMP pathway and genes involved in cardiac development [46]. PCGF4/BMI1 has been identified as a tumor suppressor that represses the Ink4a/Arf gene locus directly in collaboration with c-MYC in a transgenic model of a mouse lymphoma [57,58,70,71].

Our experiments yielded important insights into the composition of PCGF–PRC1 assembly complexes and linked alternative PRC1-related complexes to distinct molecular functions. Importantly, we showed a distinctive link between PCGF4/BMI1 and p16 expression, potentially linking this protein (and hence the PCGF4/BMI1–PRC1 complex) to the process of senescence. In line with previous studies, unique complex components have also been identified for different PCGF homologs, which suggests that they are not completely redundant and that they may also have some independent functions [15,43].

Overall, our results provide a comprehensive analysis among three different PCGF subunits containing a certain degree of protein homology. We uncovered their role in the formation of distinct PRC1 interactomes, and their functional specificities in a different cell (NT2) model. Further insights into the genome-wide localization and complex composition of variant PRC1 complexes in different cellular contexts will likely add to our understanding of their individual and overlapping functions and contribute to our understanding of the PRC1 assembly in detail. Thus far, the notion that sequence homology implies functional similarity through common interactor partners is still not proven. Furthermore, how protein similarity may influence the organization of a chromatin re-modeler has still not been elucidated. Our investigation showed even though the PCGF “family” conserved a certain protein homology, their interactome exhibited unique features. This observation suggests that differences across PCGF interactomes lead to an impact on the biological role of each PCGF protein and subsequently influence the chromatin architecture. Furthermore, systematic investigations of each PRC1 subcomplex (PRC1.1–1.6) under native conditions in the different cell lines may help in unlocking the complicated network of PRC1 interactors and understanding their precise role in cell biology and cancer progression in the future.

## 4. Materials and Methods

### 4.1. Cell Culture

NTera-2/cloneD1 (NT2) cells (ATCC, CRL-1973) were cultured and passaged in 92 mm tissue culture dishes Nunclon (Thermo Fisher Scientific, Waltham, Massachusetts, USA) in Dulbecco’s modified Eagle medium (DMEM) supplemented with 10% (*v*/*v*) fetal bovine serum (Hyclone, USA), 100 U/mL penicillin, and 100 U/mL streptomycin (Gibco, USA), as described [32].

### 4.2. Isolation of Nuclei

NT2 nuclei were isolated as described [32]. In brief, NT2 cells were harvested, washed with PBS, and resuspended in lysis buffer (25 mM Tris-HCl pH 7.6, 150 mM NaCl, 1% Nonidet P-40, 1% sodium deoxycholate, 0.1% SDS, 2 μg/mL aprotinin, 1 μg/mL, leupeptin, and 10 mM PMSF). The lysates were incubated for 15 min on ice and the cell membrane was disrupted mechanically by syringing with a 21 G narrow gauge needle, then the sonicated lysates were incubated on ice for 15 min and cleared by centrifugation at 20,000× *g* at 4 °C for 30 min. To harvest the nuclear fraction, lysates were resuspended in an equal volume of nuclear buffer (20 mM HEPES pH 7.9, 0.2 mM EDTA, 1.5 mM MgCl_2_, 20% glycerol, 420 mM NaCl, 2 μg/mL aprotinin, 1 μg/mL leupeptin, and 10 mM PMSF) and dounced with pestle type B. Lysates were incubated for 45 min on a rotator at 4 °C to dissociate chromatin-bound proteins and precleared by centrifugation at 20,000× *g*, 4 °C for 30 min.

### 4.3. Immunoprecipitation

Immunoprecipitations (IPs) were performed on the nuclear protein lysates prepared in IP buffer, as described [32]. Briefly, 10 μg of antibody was coupled to 50 μL packed Protein A beads (Sigma P9424) by incubation in 1 mL PBS (0.1% Tween-20) at 4 °C with rotating overnight. Beads were collected by centrifugation at 1700× *g* for 3 min and washed twice in 1 mL 0.2 M sodium borate, pH 9.0. Antibodies were then crosslinked to beads and the reaction was terminated with 1 mL of 0.2 M ethanolamine, pH 8.0, then incubated for 2 h at room temperature on a rotator. Beads were washed twice in Buffer C100 (20 mM HEPES pH 7.6, 0.2 mM EDTA, 1.5 mM MgCl_2_, 100 mM KCl, 0.5% Nonidet P-40, 20% glycerol). Antibody-crosslinked beads were incubated with nuclear lysates in the presence of 250 U/mL benzonase nuclease, at 4 °C rotating overnight, and washed five times in Buffer C100 with 0.02% Nonidet P-40. After the final wash, beads destined for immunoblotting were resuspended in 50 μL 2× SDS sample buffer and boiled for 5 min under agitation to elute the proteins. The eluent was run on SDS-PAGE and analyzed by immunoblotting. The beads destined for mass spectrometry analysis were washed once in IP buffer containing 0.02% Nonidet P-40, followed by one wash in the IP buffer with no detergent.

### 4.4. Mass Spectrometry Analysis

Proteins were treated with trypsin as described [32,72]. Samples were resuspended in 50 μL of trifluoroacetic acid 0.1% (*v*/*v*) in water, as buffer A, sonicated for 1 min, and centrifuged for 15 min at 15,000× *g*. Analysis was carried out on an Ultimate 3000 RSLCnano HPLC system connected to a Q Exactive, high-resolution, and mass accuracy, mass spectrometer (Thermo Fisher Scientific, Waltham, MA, USA). The MS instrument was controlled by Xcalibur software (Thermo Fisher Scientific, Waltham, MA, USA). Each sample was loaded onto a 75 µm × 15 cm C18 column (particle diameter 1.8 µm, pore size 120 Å) and separated by an increasing acetonitrile gradient over 100 min at a flow rate of 200 nl/min. MS analysis was conducted in DDA mode: parent ion spectra (MS1) were measured at a resolution of 60,000, AGC target 3e6. Tandem mass spectra (MS2; up to 20 scans per duty cycle) were obtained at a resolution of 15,000, AGC target 2e5, and collision energy of 27.

### 4.5. Data Processing

Data were processed using MaxQuant version 1.4.3.22 [73] using the human UniProt database (Taxon identifier 9606, Proteome ID 630 UP000005640, Protein Reviewed 20,380). The following search parameters were used: Fixed Mod: carbamidomethylation; Variable Mods: methionine, oxidation; Trypsin/P digest enzyme (maximum two missed cleavages); Precursor mass tolerances, 6 ppm; Fragment ion mass tolerances, 20 ppm; Peptide FDR 1%; Protein FDR 1%.

“Label-Free Quantitation; LFQ”, “iBAQ”, and “Match Between Run” settings were selected. Reverse hits and contaminants retrieved from the cRAP database (https://www.thegpm.org/crap/, assessed on 1 January 2024) [74] were filtered out and not considered further.

### 4.6. Data and Statistical Analysis

Bioinformatic analysis of the MaxQuant output files and data visualization was performed with Perseus software version 1.4 [75] and RStudio by employing the following packages: ggplot2, ggrepel, and clusterProfiler. LFQ values were extracted from the protein group (Supplementary Material Table). No additional normalization steps were performed as the resulting LFQ intensities were normalized by the MaxLFQ procedure [76]. In the Perseus software, the LFQ values were transformed (log2) and a protein was considered quantified only if it was detected in at least two out of three biological replicates. Missing value imputation was carried out from a normal distribution (width: 0.3, downshift: 1.8), and a two-tailed *t*-test was applied with correction for multiple testing (Benjamini). Volcano plots were constructed using the permutation-based FDR (1%) approach [75,77] and we set the significant differences in the protein abundance (≥1.5-fold change). Gene Ontology analysis was performed using the ‘enrichGO’ function of the cluster Profiler R and Bioconductor package with the parameters ‘pAdjustMethod = ‘BH’, ont = ‘BP’, q value cutoff = 0.05 [78]. The share and distinctive interactome dataset were independently analyzed against the *“homo sapiens”* (org.Hs.eg.db) genome background using the default setting.

Protein alignment was performed using Clustal-Omega with the default settings (https://www.ebi.ac.uk/Tools/msa/clustalo/, assessed on 1 January 2024) [36]

### 4.7. Immunoblotting

Protein lysate was quantified using the Bradford assay. Subsequently, protein lysates were separated on SDS–PAGE and transferred to nitrocellulose membranes. Membranes were blocked with 5% non-fat milk or 5% BSA at room temperature for 1 h and incubated overnight with diluted primary antibody at 4 °C. Membranes were then washed and incubated with the HRP-conjugated goat anti-rabbit or mouse IgG secondary antibody for 1 h at room temperature. The membrane was incubated with enhanced chemiluminescence reagents (Thermo Scientific, USA) followed by exposure to X-ray films. Immunoblotting was performed using the antibodies and conditions listed in the Supplementary Materials.

### 4.8. Gel Filtration Column Chromatography

The SuperoseTM 6 10/300 GL gel filtration column (GE Healthcare) was equilibrated with one column volume of running buffer (20 mM Tris pH 8.0, 10% glycerol, 175 mM NaCl, 0.5 mM DTT, and 1 mM PMSF). Around 300–500 μg of total nuclear protein (prepared as described above) was injected and run through the column at 0.35 ml/min. The 1 mL fractions were collected, and the protein was concentrated by incubation with 4 μL StrataClean resin (Agilent Technologies, Santa Clara, CA, USA) for 1 h at room temperature. The resin was collected by centrifugation at 2500× *g* for 3 min, and the protein was eluted by boiling in 20 μL 2X SDS sample buffer for 5 min under shaking. The eluted protein was analyzed by SDS-PAGE and immunoblotting.

### 4.9. Real-Time Quantitative PCR

Extracted RNA was used to generate cDNA by reverse transcriptase PCR using the TaqMan Reverse Transcription Kit (Applied Biosytems, Waltham, MA, USA). Relative mRNA expression levels were determined using the SYBR Green I detection chemistry on the LightCycler 480II Real-Time PCR System (Roche, Welwyn Garden City, UK). The ribosomal constituent RPO was used as a normalizing gene. The primers used are listed in the Supplementary Materials.

### 4.10. Cell Viability Assay

Lentivirus-infected NT2 cells were subjected to crystal violet staining using 0.1% crystal violet (CV) in a microtiter plate. After staining, wells were washed with phosphate-buffered saline (pH = 7.4) to remove the unbound crystal violet and residual NT2 cells. The plates were then air-dried at room temperature, and 95% ethanol was added to the wells to resuspend the adhered stained cells. The ethanol-bound crystal violet stains of the adhered cells were quantified by measuring at 590 nm on a spectrophotometer.

## 5. Conclusions

The investigation of the interactome landscape appears to be a very important distinguishing factor in the definition of the degree of protein similarity in protein families. The integration of protein homology analysis and affinity purification, followed by mass spectrometry analysis, may open a new avenue in solving one of the central problems in modern biology: the aim of identifying the complete set of protein interactions in, and the important biological processes of a cell including catalyzing metabolic reactions, DNA replication and transcription, response to stimuli, and transporting molecules from one location to another.

## Data Availability

The original contributions presented in the study are included in the article/Supplementary Materials, further inquiries can be directed to the corresponding authors.

## Supplementary Materials

The supporting information can be downloaded at: https://www.mdpi.com/article/10.3390/ijms25189809/s1.
