# Supplementary material for: PRC1 Protein Subcomplexes Architecture: Focus on the Interplay between Distinct PCGF Subunits in Protein Interaction Networks
Source: Int J Mol Sci. 2024 Sep 11;25(18):9809. doi: 10.3390/ijms25189809 (PMC11432245; doi:10.3390/ijms25189809)
Supplement: Supplementary file 1 [file ijms-25-09809-s001.zip › Supplementary Material Figures.pdf]

Supplementary Material: Figures S1 and S2

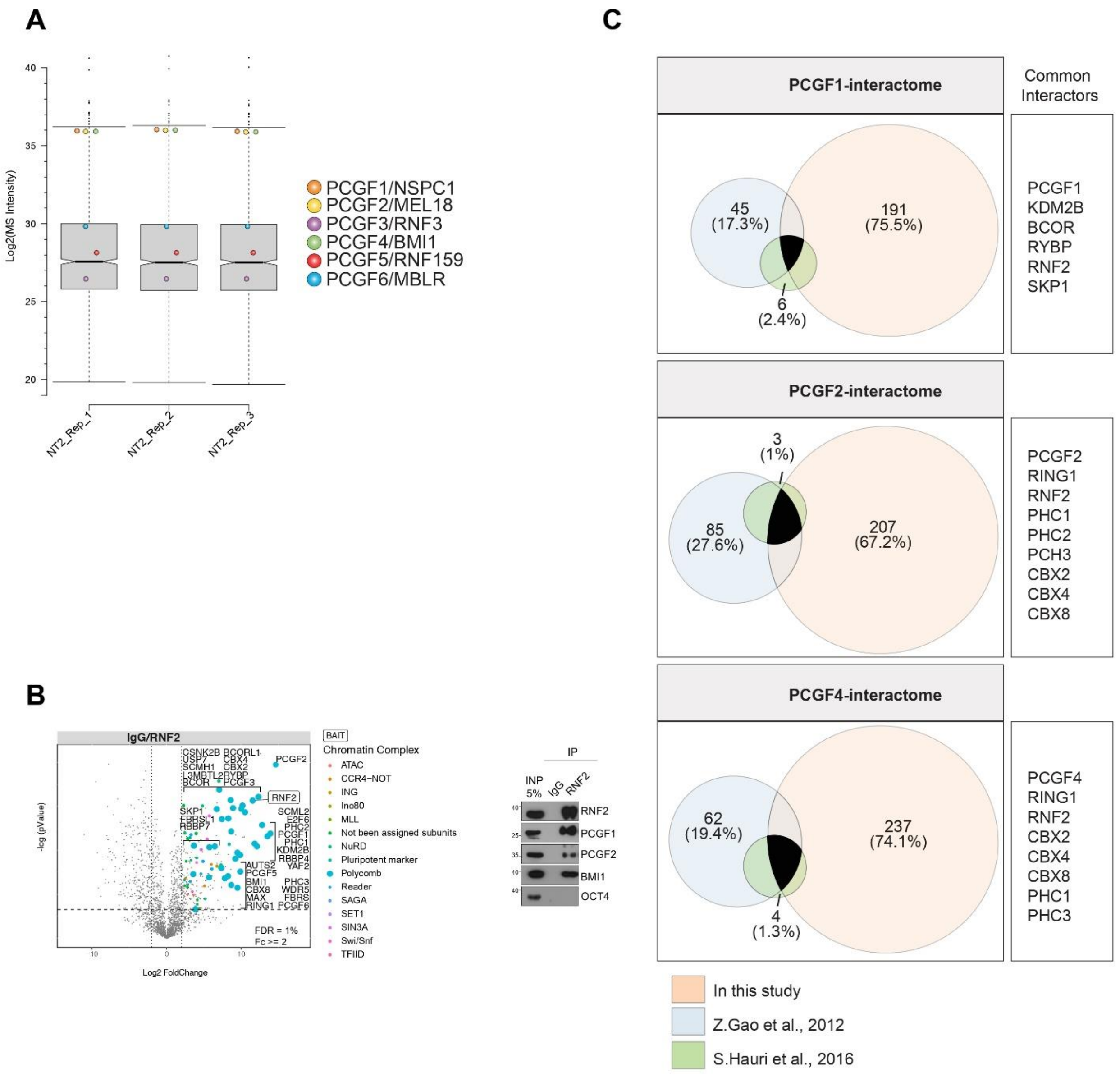

__**Figure S1: AP-MS PCGFs screening analysis in NT2 cells.**__

A) Box plots of protein expression profile in NT2 cell line. The normalized MS intensity signal for each protein (LFQ intensity) shows the relative PCGFs abundance profile compared to the whole proteome. B) The set of identified proteins for the RNF2 immunoprecipitation experiment was projected onto volcano plots to identify statistically robust hits. The specificity of the antibodies used in the RNF2 immunoprecipitation was confirmed using western blotting. PCGF1,

PCGF2 and PCGF4 co-immunoprecipitated in RNF2 immunoprecipitation. C) Venn diagram showing the range of overlap among the physical interactomes of PCGF1, PCGF2, and PCGF4 among our study compared to previous studies by Gao and Hauri *et al* (45 & 46).

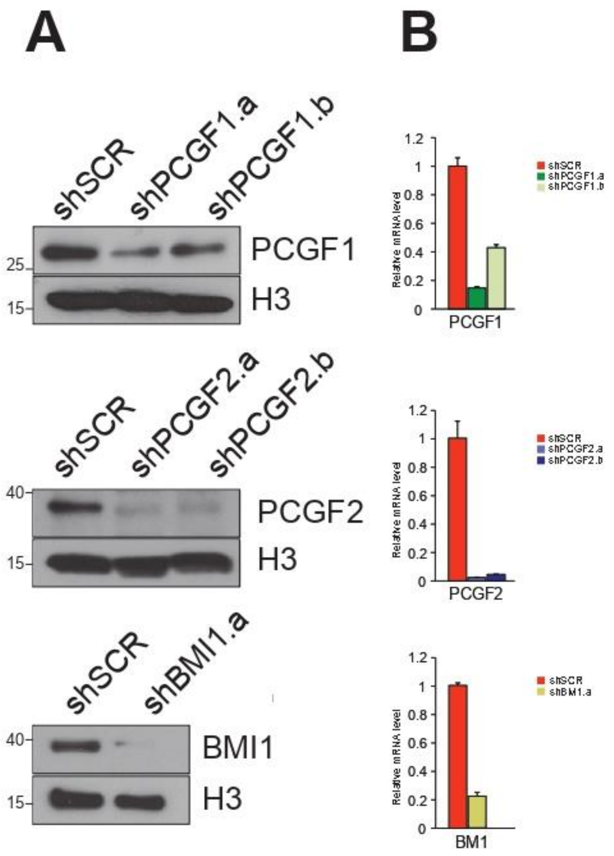

<u>Figure S2: Functional PCGFs shRNA screening in HEK293T</u>.

A) Western blotting of HEK293T cells transfected with different PCGF shRNA vectors.

Nuclear protein lysates were analysed by Western blotting.

B) Efficiency of shRNA vectors were analysed by qRT-PCR.
