# Supplementary material for: PRC1 Protein Subcomplexes Architecture: Focus on the Interplay between Distinct PCGF Subunits in Protein Interaction Networks
Source: Int J Mol Sci. 2024 Sep 11;25(18):9809. doi: 10.3390/ijms25189809 (PMC11432245; doi:10.3390/ijms25189809)

Supporting Information Fig:2

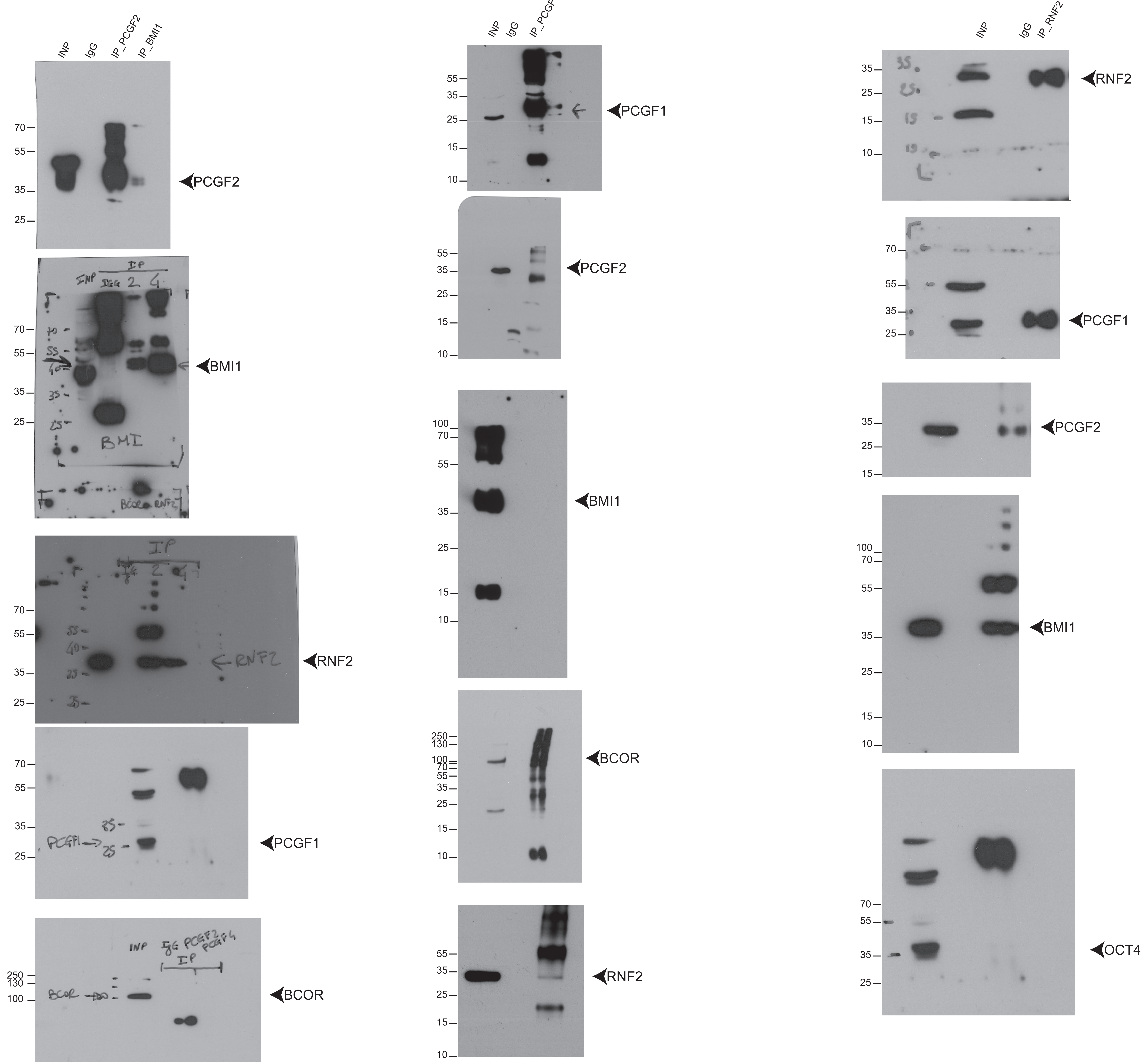

Supporting Information Fig:3

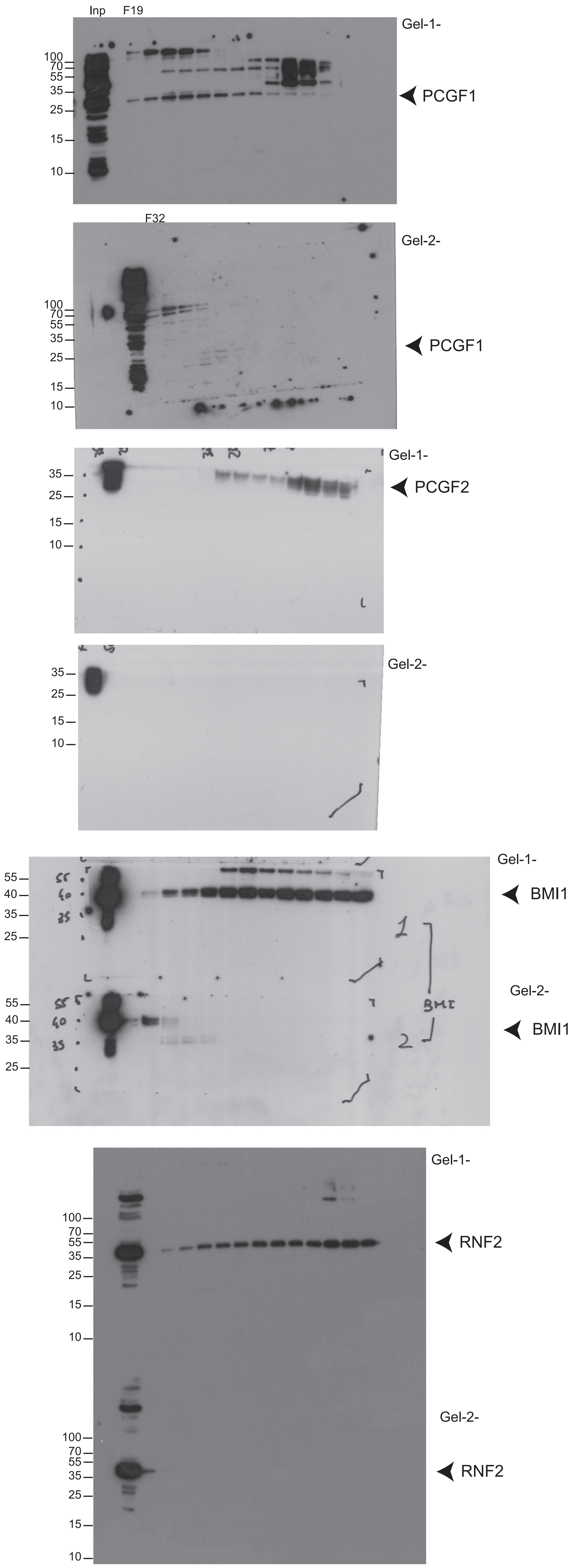

SDS-PAGE [15% (w/v)
acrylamide resolving gel]

Supporting Information Fig:5

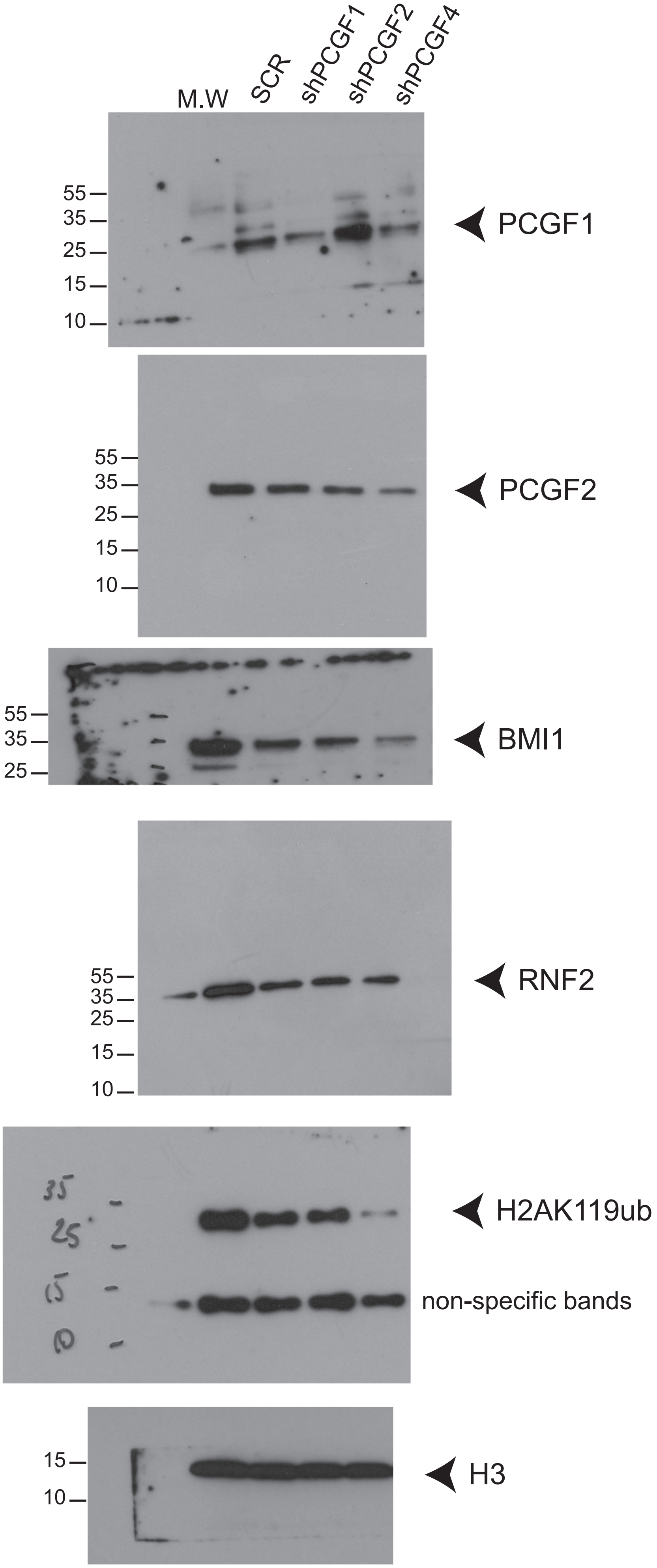

Supporting Information Fig:S1

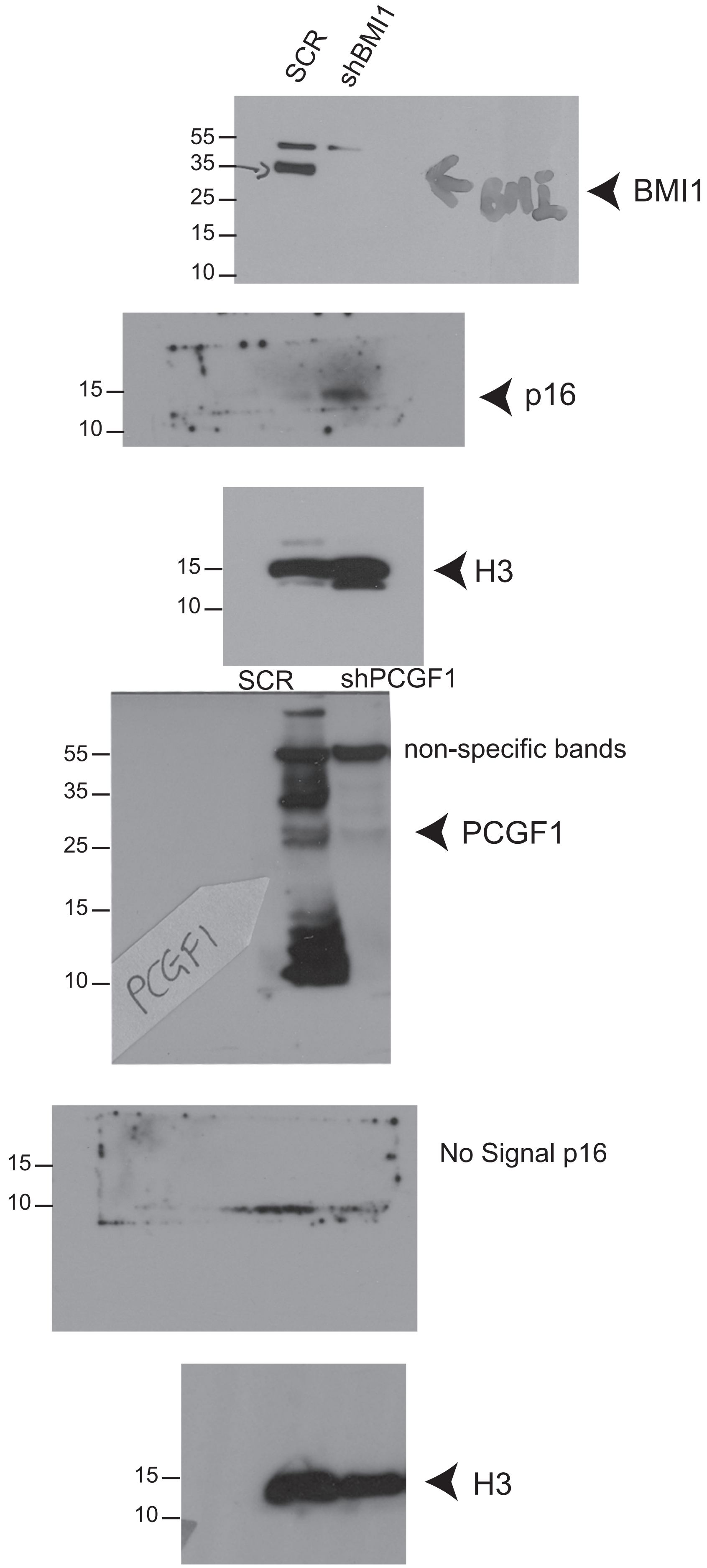

Supplement: Supplementary file 1 [file ijms-25-09809-s001.zip › Validation of figures 2,3,5 and S1.pdf]
